# Comparative analysis of the kinomes of *Plasmodium falciparum*, *Plasmodium vivax* and their host *Homo sapiens*

**DOI:** 10.1186/s12864-022-08457-0

**Published:** 2022-03-26

**Authors:** Jack Adderley, Christian Doerig

**Affiliations:** School of Health and Biomedical Sciences, RMIT University, 3083 Bundoora, VIC Australia

**Keywords:** Malaria, *Plasmodium falciparum*, *Plasmodium vivax*, Kinome, Kinase

## Abstract

**Background:**

Novel antimalarials should be effective across all species of malaria parasites that infect humans, especially the two species that bear the most impact, *Plasmodium falciparum* and *Plasmodium vivax*. Protein kinases encoded by pathogens, as well as host kinases required for survival of intracellular pathogens, carry considerable potential as targets for antimalarial intervention (Adderley et al. Trends Parasitol 37:508–524, 2021;  Wei et al. Cell Rep Med 2:100423, 2021). To date, no comprehensive *P. vivax* kinome assembly has been conducted; and the *P. falciparum* kinome, first assembled in 2004, requires an update. The present study, aimed to fill these gaps, utilises a recently published structurally-validated multiple sequence alignment (MSA) of the human kinome (Modi et al. Sci Rep 9:19790, 2019). This MSA is used as a scaffold to assist the alignment of all protein kinase sequences from *P. falciparum* and *P. vivax*, and (where possible) their assignment to specific kinase groups/families.

**Results:**

We were able to assign six *P. falciparum* previously classified as OPK or ‘orphans’ (i.e. with no clear phylogenetic relation to any of the established ePK groups) to one of the aforementioned ePK groups. Direct phylogenetic comparison established that despite an overall high level of similarity between the *P. falciparum* and *P. vivax* kinomes, which will help in selecting targets for intervention, there are differences that may underlie the biological specificities of these species. Furthermore, we highlight a number of *Plasmodium* kinases that have a surprisingly high level of similarity with their human counterparts and therefore not well suited as targets for drug discovery.

**Conclusions:**

Direct comparison of the kinomes of *Homo sapiens*, *P. falciparum* and *P. vivax* sheds additional light on the previously documented divergence of many *P. falciparum and P. vivax* kinases from those of their human host. We provide the first direct kinome comparison between the phylogenetically distinct species of *P. falciparum* and *P. vivax*, illustrating the key similarities and differences which must be considered in the context of kinase-directed antimalarial drug discovery, and discuss the divergences and similarities between the human and *Plasmodium* kinomes to inform future searches for selective antimalarial intervention.

**Supplementary Information:**

The online version contains supplementary material available at 10.1186/s12864-022-08457-0.

## Background

Despite significant progress in reducing malaria morbidity and mortality over recent decades, the disease remains a major health burden in tropical and subtropical regions, and puts significant strain on the medical and economic systems of heavily affected countries [1]. Malaria is caused by parasites of the genus *Plasmodium*, with *Plasmodium falciparum* and *Plasmodium vivax* being the most clinically relevant species. The World Health Organisation’s current goal to achieve global elimination of malaria requires significant support for continued research and development into novel drugs with untapped targets to support the failing frontline treatments of the disease [2]. All currently deployed antimalarials are facing various levels of parasite resistance, which threatens to erode global progress towards eradication [3]. This is further marred by the lack of a highly efficacious vaccine (despite recent encouraging progress in this area) and by the ongoing SARS-CoV-2 pandemic that diverts public health resources away from malaria and other tropical diseases [4–6].

This dire situation calls for the development of novel control agents with untapped modes of action that should be effective against both *P. falciparum* and *P. vivax*. One key class of signalling molecules, protein kinases, have been successfully targeted to treat numerous diseases; for example, 62 kinase inhibitors have reached the market for the treatment for a variety of conditions (prominently cancer), and this number keeps increasing [7]. Protein kinases encoded by pathogens, as well as host kinases required for survival of intracellular pathogens, carry considerable potential as targets for the treatment of infectious diseases such as malaria [8, 9]. Protein kinases catalyse the transfer of phosphate from adenosine triphosphate (ATP) to a substrate polypeptide, resulting in structural and functional changes to the target protein. Phosphorylation events orchestrated by kinases in tandem with protein phosphatases play a pivotal role in signalling in all living cells. Many protein kinases are highly conserved even among evolutionarily distant organisms [10]. The kinome (i.e. the complement of all protein kinase-encoding genes) of *P. falciparum* was first assembled in 2004, reporting a total of 85 protein kinases, 65 of which, were related to typical eukaryotic protein kinases (ePKs) [11]. ePKs share a conserved catalytic domain, which contains 12 distinct subdomains/motifs [12]. A majority of ePKs can be assigned to one of the following groups: CAMK, CMGC, AGC, TKL, TK, CK1 and RGC; ePK sequences that do not cluster in any of these groups by phylogenetic analysis comprise the “OPK” (other protein kinase) group; this includes important families such as the NEKs [13]. In addition to ePKs, proteins that do not possess the 12 aforementioned ePK domains but display kinase activity are grouped in a number of “atypical kinase” (aPK) families (see below). Over the past decades, the number of protein kinases identified in *P. falciparum* has steadily increased with the most recent study indicating a total of 105 protein kinases, (98 typical protein kinases and 7 atypical protein kinases, aPKs) [8, 14]. Interestingly, the kinome of *Plasmodium vivax* has received considerably less attention, with, to our knowledge, only a brief overview available in the literature [15], with no formally available phylogenetic tree of the parasite’s protein kinases or detailed comparison with the *P. falciparum* kinome; we intend to fill in this gap through the present study.

We utilised a recently published structurally-validated multiple sequence alignment (MSA) of the human kinome [16] as a scaffold to assist the alignment of all protein kinase sequences from *P. falciparum* and *P. vivax*, and (where possible) their assignment to specific kinase groups/families. Through this strategy we were able to assign six *P. falciparum* previously classified as OPK or ‘orphans’ (i.e. with no clear phylogenetic relation to any of the established ePK groups) to one of the aforementioned ePK groups (see below). The kinomes of *Homo sapiens*, *P. falciparum* and *P. vivax* were directly compared, shedding additional light on the previously documented divergence of many kinases, as expected in view of the phylogenetic distance between Opisthokonts and Alveolates (the clades that include metazoans and *Apicomplexa*, respectively), as well as new evidence for the conservation of a handful of kinases across both Opisthokonts and Alveolates. We also provide the first direct kinome comparison between the phylogenetically distant species of *P. falciparum* and *P. vivax*, illustrating the key similarities and differences which must be considered in the context of kinase-directed antimalarial drug discovery.

## Results and discussion

### Protein kinase domain identification and assembly

The *P. falciparum* sequences were collected from our earlier alignment [8], while *P. vivax* sequences were obtained by searching the predicted *P. vivax* proteome in PlasmoDB v50b [17] using the term “kinase”. The resultant list was further refined to only include (i) sequences containing a Pfam ID of PF00069 or PF07714 (Protein kinase domain and protein tyrosine kinase, respectively) and (ii) sequences which contained the phrase “protein kinase” in ‘Product description’ data. These sequences were assessed using ScanProsite [18] to identify the protein kinase domain. Two regulatory subunits of CK2 (CK2β, both of which have characterised orthologues in *P. falciparum* [19]), a sequence annotated as “putative protein kinase” but that did not have a recognisable protein kinase domain, and a protein phosphatase, were removed (PVP01_0904500, PVP01_1212400, PVP01_1030400 and PVP01_1406400). Four atypical protein kinases (aPK) were identified: ABCK1 and ABCK2 from the ABC family (PVP01_1430100, PVP01_1334400), and Rio1 and Rio2, (PVP01_1449100, PVP01_ 0529500) all of which have orthologues in *P. falciparum* [11]. Finally, we identified a surprising four members of the phosphatidylinsositol kinase (PIK) family in *P. vivax*, whereas this family is represented by only three enzymes in *P. falciparum*. It would be interesting to determine if this additional PIK family enzyme (PVP01_1309200) is implicated in *P. vivax*-specific biology, e.g. preference for reticulocyte or ability to establish dormant forms (hypnozoites) in hepatocytes. Although transcriptomics studies compiled on PlasmoDB suggest that the gene seems to be transcribed throughout the erythrocytic asexual cycle [20], expression of the gene appears to be lower in the hypnozoite-enriched fraction than in samples from mixed (non-enriched) hepatic schizonts [21] (it may be of interest to determine whether expression is resumed once the hypnozoite reactivates). These sequences are PVP01_1018600 (orthologous to PfPIK3), PVP01_1024200 (orthologous to PfPIK4), PVP01_0529300 (orthologous to PF3D7_0419900) and PVP01_1309200. Phylogenetic analysis of the PIKs kinase domains indicated that PVP01_1309200 is divergent (Supplementary Fig. 1). A fifth *P. vivax* PIK-like kinase was initially identified (PVP01_1404700), but its low Hidden Markov Model (HMM) score < 50 (as defined by HMMER 3.3 [22]), and subsequent manual examination of the sequence, indicated this protein is highly divergent from the consensus PIK sequence and hence was not included here. We further sought to determine if any additional protein kinases were encoded by *P. vivax* through a Psi-BLAST search (PlasmoDB’s Beta Blast interface – multiple parallel blast searches). We included the protein kinase domains of *P. vivax* (identified here), and those of *P. falciparum* [8] in the search query; however, no significant additional sequences were identified in this way. The typical protein kinase domains (78 in *P. vivax* [up from the 68 reported in the aforementioned preliminary study [15], 98 in *P. falciparum*) were initially aligned using Clustal Omega [23] and imported into Jalview [24] for manual alignment adjustments.

### Hidden Markov Model (HMM) profiling for assignment of sequences to ePK families

To assist the development of a multiple sequence alignment (MSA) of the *Plasmodium* protein kinase domains, each sequence was assessed using HMMER 3.3 [22] using the defined kinase families reported in Kinomer V1.0 [25]. Kinomer contains a profile for the AGC, CAMK, CK1, CMGC, RGC, STE, TKL and TYR ePK groups, and also includes a profile for the apicomplexan specific FIKK family [11, 26], but does not have a profile for the NEK family (traditionally considered to belong to the OPK group [see above]). In view of the importance of NEKs in all eukaryotes [27] including malaria parasites [28], we designed a NEK profile with 21 known kinases from the NEK family [29] (see Methods for details). Using the amended Kinomer library (now containing a NEK profile), we performed a HMMER scan (hmmscan) of the kinomes and designated each sequence with the top hit based on score and E-value (see Supplementary data 1 for full hmmscan results). Each sequence was assessed to determine if it met the e-value thresholds for group assignment [25]. These threshold values differ for each group according to the highest E-value obtained during each groups hmm profile construction AGC (2.7e^− 7^), CAMK (3.2e^− 14^), CK1 (3.2e^− 5^), CMGC (1.2e^− 7^), RGC (4.8e^− 5^), STE (1.4e^− 6^), TK (1.1e^− 9^), TKL (1.7e^− 12^) (see [25] for details). To allow for a conservative group assignment we also considered assignments with a bit score < 50 to be unreliable. This conservative value is double the default bit score threshold of the online HMMER tool www.ebi.ac.uk/Tools/hmmer/. Phylogenetic trees with the *H. sapiens*, *P. falciparum* and *P. vivax* sequences for each of the ePK groups are available as Supplementary Figs. 2, 3, 4, 5, 6, 7 and 8.

### Multi-sequence alignment using the human kinome as a scaffold

**Fig. 1:**
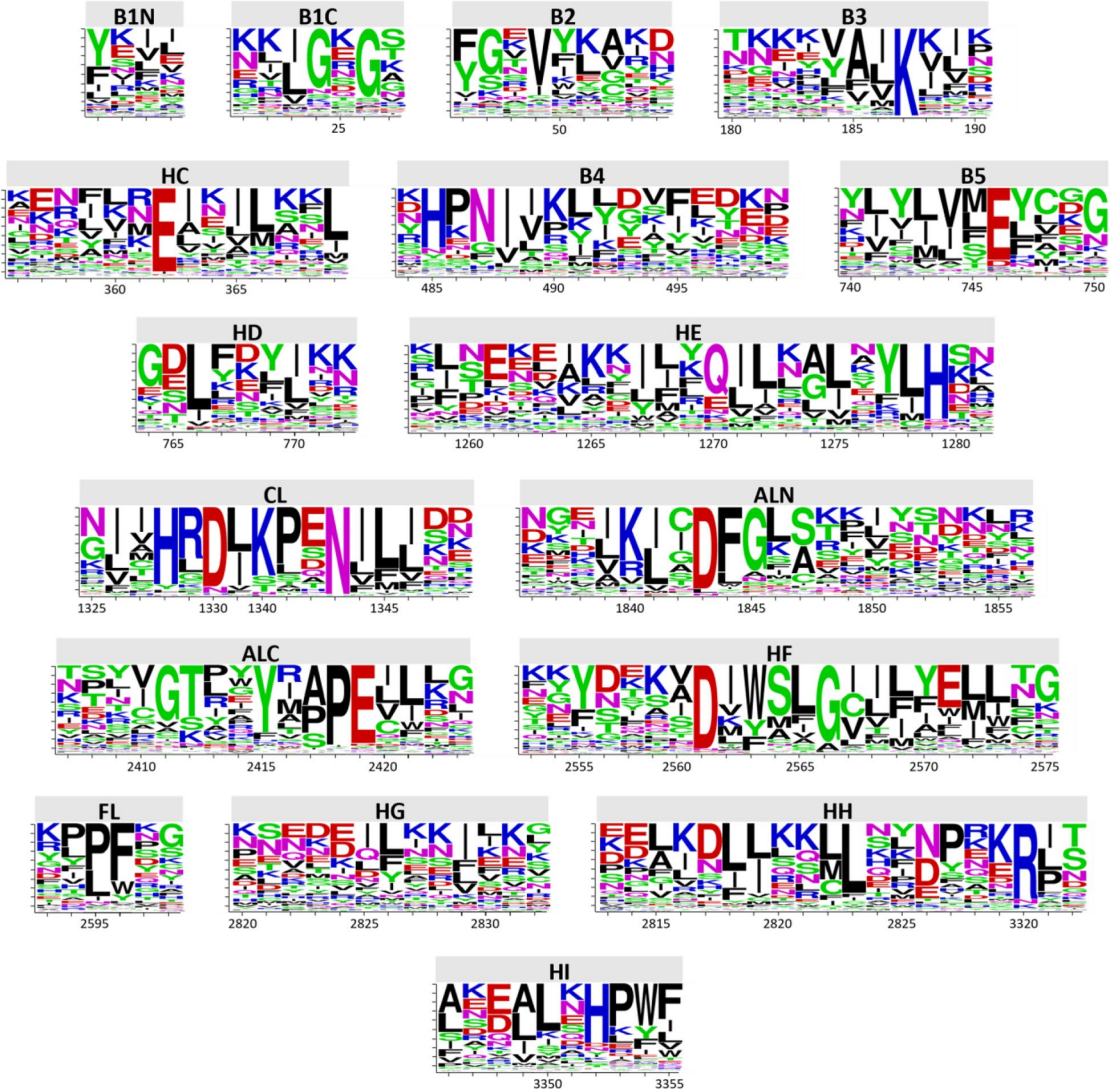
Sequence logos of the conserved regions in the multisequence alignment for the kinase domains of *P. falciparum* and *P. vivax*. Aligned regions of the kinase domain defined by [38] and logo generated using the webserver WebLogo (https://weblogo.berkeley.edu/) [39] and edited in Adobe Illustrator

The human kinome comprises approximately 478 typical protein kinases, which contain a total of 497 typical protein kinase domains (as some sequences have two kinase domains) [16]. Of the 497 protein kinase domains known, over 270 have had their crystal structure solved. This is in stark comparison to the low number of solved protein kinase domains of *P. falciparum* and *P. vivax* (to date, 8 and 1 respectively). For *P. falciparum* these kinase domains are from PK5 [30], PK7 [31], PKG [32], CDPK3 [33], CDPK4 (PDB: 4RGJ), MAPK2 (PDB: 3NIE), CK2 [34] and CLK1 (PDB: 3LLT); for *P. vivax* only PKG [32]. The large number of kinase domain structures available supported the human kinome MSA, which was not possible for the *P. falciparum* and *P. vivax* kinomes. We therefore leveraged the homology between kinase families across species to aid in the MSA for both *P. falciparum* and *P. vivax* kinase domains (see Methods). The 17 conserved segments (230 amino acids) used for the alignment (see Methods) are depicted in Fig. 1 as sequence logos for the *Plasmodium* kinases, to highlight the conserved motifs important for kinase function from within the domain. Key motifs are (i) His-Arg-Asp (HRD), which is within the catalytic loop and stabilises the active site [35], (ii) Asp-Phe-Gly (DFG), also within the activation loop and mediates allosteric conformational changes to regulate activation/inactivation of the enzyme [36], and (iii) Ala-Pro-Glu (APE), which sits at the C-terminal end of the activation loop and stabilises the segment through docking to the domains F-helix [37]. The HRD, DFG and APE motifs are part of the conserved segments in the catalytic loop (CL), Activation loop N-terminal (ALN) and Activation loop C-terminal (ALC), respectively (see Fig. 1). To determine if the overall conserved segments of the kinase domain differ between *Plasmodium* and *Homo sapiens*, we generated sequence logos for the *Homo sapiens* sequences as well (Supplementary Fig. 9). No large differences can be detected, but the consensus sequence is not as pervasive across the *Plasmodium* kinases, suggesting *Plasmodium* kinases can accommodate less stringent constraints at the primary structure level. However, this could be an artefact due to the kinomes of *Plasmodium* being smaller than that of humans, resulting in more divergent kinases making up a greater proportion of the kinome.

### Phylogenetic tree of the *Homo sapiens, P. falciparum and P. vivax kinomes*

**Fig. 2:**
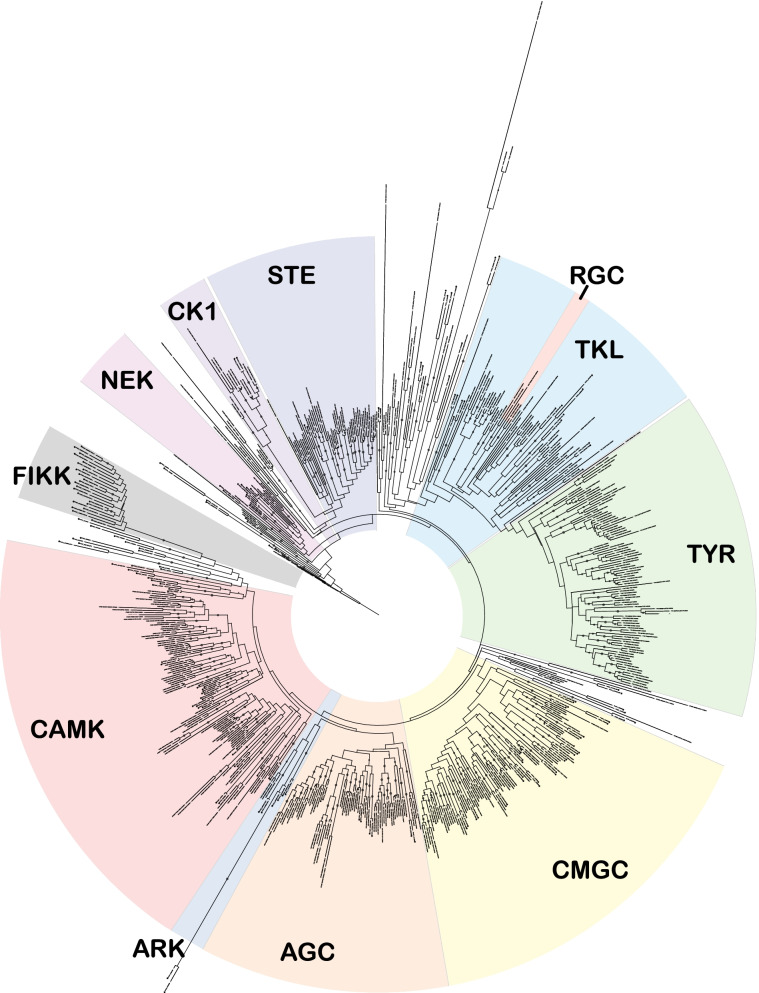
Phylogenetic tree containing the protein kinome of *Homo sapiens, Plasmodium falciparum, Plasmodium vivax*. The tree is represented in a circular format and contains a total of 671 protein kinases sequences excluding the atypical kinases (H. sapiens − 497, *P. falciparum* − 98 and *P. vivax* – 78). *P. falciparum* sequences were accessed from [8], *H. sapiens* sequences were accessed from [16] and *P. vivax* sequences were identified from PlasmoDB [17] and initially aligned using ClustalOmega [23]. All of the kinase sequences were imported into Jalview [24]. Using the human kinases as a template. the *Plasmodium* kinases were aligned into the conserved regions as defined by [16]. The resultant 230 column alignment was assessed using RAxML to infer phylogenetic distances and determine bootstrapping [41]. RAxML Gui 2.0 [42] was used with the following parameters: maximum-likelihood rapid bootstrap with the PROTgamma substitution model LG4M, with AutoMRE. A gene tree was inferred through the RAxML analysis using the ‘best tree’ and rendered with the interactive tree of life webserver (iTOL) [44]. HMMER profiling was performed for *P. falciparum* and *P. vivax* kinases using the defined families of Kinomer [25] with the addition of the NEK family (see Supplementary data 3). Using the tree structure, HMMER results and the known human kinase family assignments the *Plasmodium* kinases were assigned to the 9 typical protein kinase groups. These family assignments were annotated using Adobe illustrator along with the Aurora kinase family (ARK) and the Apicomplexan-specific kinase family FIKK. Orphan, or ‘other’ kinases are largely unassigned to families (white background). Bootstraps values above 50 are represented as circles on the associated branches, larger circles indicate higher bootstrap values. *Plasmodium* kinases are indicated with a red star and, the associated branches are bold

**Fig. 3:**
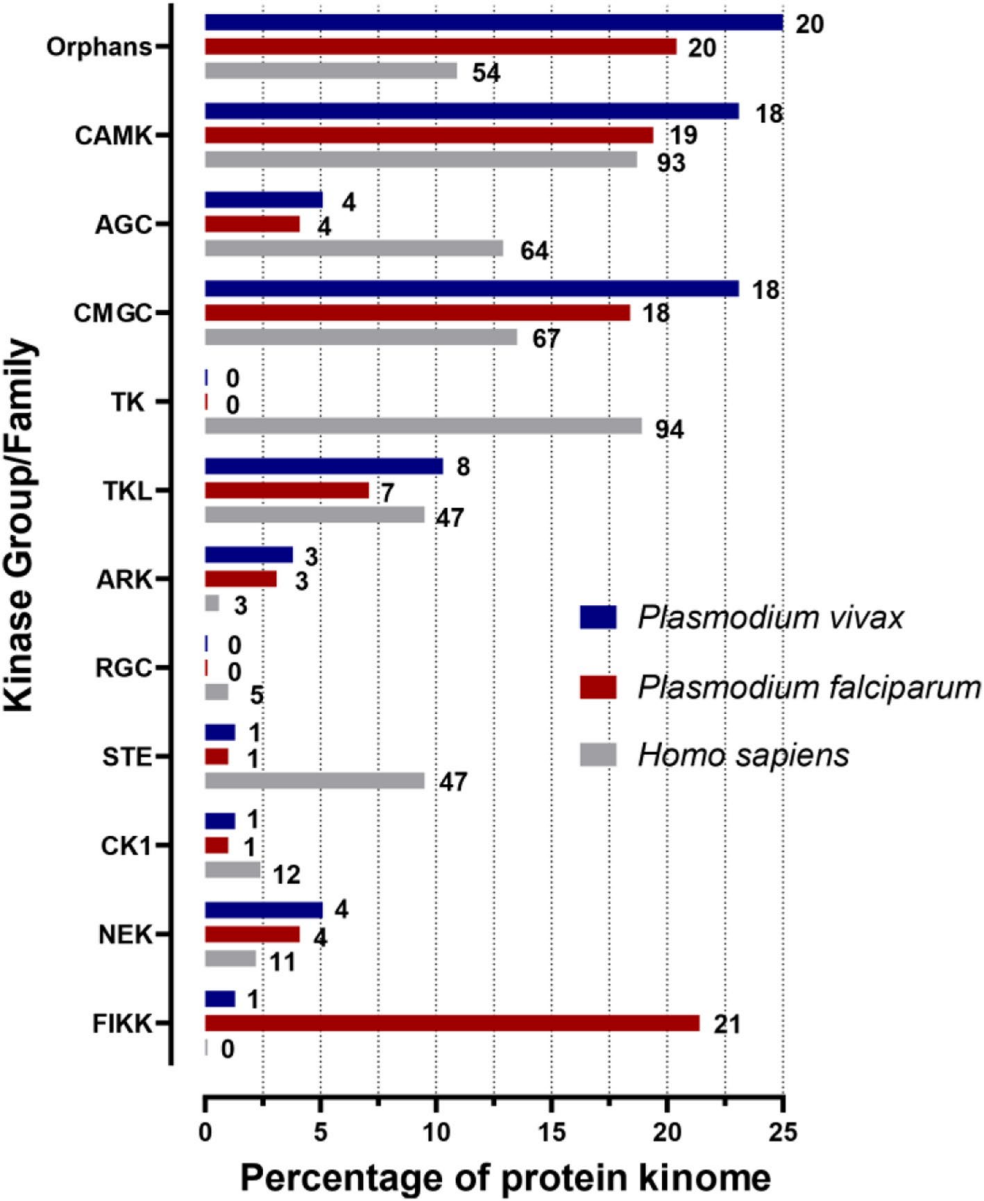
Visualisation of the protein kinase family membership across *Homo sapiens, Plasmodium falciparum, Plasmodium vivax*. The nine typical protein kinase families along with the Aurora kinase family (ARK) and the Apicomplexan-specific family of FIKK were included here. The remaining unassigned kinases are denoted as Orphans. Each group/family is represented as a percentage of the total protein kinome for each organism. Note atypical protein kinases are not included in this analysis. The number next to each bar indicates the number of kinases which belong to each of the respective families for each organism. Blue = *Plasmodium vivax*, Red = *Plasmodium falciparum* and Grey = *Homo sapiens*

A phylogenetic tree (Fig. 2) was constructed from the 230-column alignment consisting of the 17 aforementioned conserved segments (see Methods), as the removal of insertions improves accuracy [40]. To determine the phylogenetic relationship between the human kinome and that of *P. falciparum* and *P. vivax*, we included a total of 671 kinase domain sequences, covering all typical protein kinase sequences from these three organisms. The phylogeny relationships were determined using the RAxML GUI 2.0 [41, 42]. The definition of the boundaries for each PK group was guided by the HMM profiles, the tree structure and the defined family assignment reported for each of the human kinases [16]. Six protein kinase previously flagged as orphans could now be confidently assigned to one of these ten groups, (see Table 1 for changes in kinase group assignment). Figure 3 depicts the number of kinases in each family per organism as a percentage of their kinome. This confirms previous reports that there are no *Plasmodium* kinases in either the TK or RGC groups [11, 14]. However, we determined that both *P. falciparum* and *P. vivax* both have a single kinase belonging to the STE family (previously only reported in *P. vivax* [38]). In addition to the STEs, a clear reduction the AGC family (in comparison to the human kinome) can be observed as well. Orphan kinases make up a much larger percentage of the kinomes of both *Plasmodium* species (as compared to the human kinome), which presumably reflects the fact that the ePK groups were historically defined using Opisthokont organisms (metazoans and yeasts). The 21-member FIKK family, discovered in the context of the initial characterisation of the *P. falciparum* kinome [11], has a single member present in *P. vivax*, consistent with the observation that the expansion of the FIKK family is restricted to parasites of the Laverania subgenus [43].

**Table 1:** Group assignment of previously orphan *P. falciparum* kinases

Kinase ID	Name	Group Assignment (this study)
PF3D7_0309200	ARK2	ARK
PF3D7_1356800	ARK3	ARK
PF3D7_1121900	-	AGC
PF3D7_0605300	ARK1	ARK
PF3D7_1145200	-	CAMK
PF3D7_1441300	-	CAMK
PF3D7_0213400	PK7	CAMK
PF3D7_0926000	-	TKL
PF3D7_0926100	-	TKL

### Plasmodium kinases with homology to human kinases

**Fig. 4:**
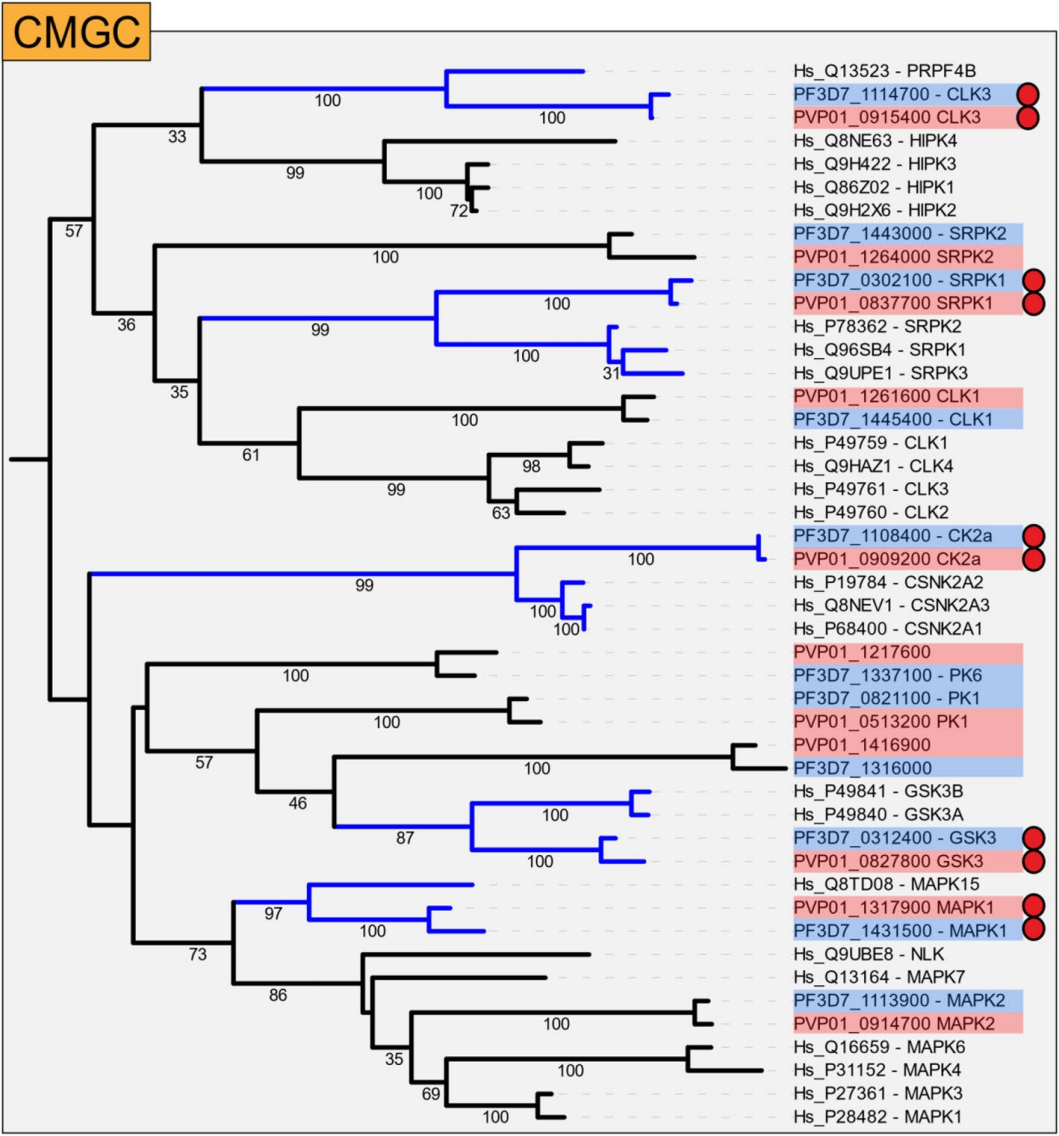
Truncated phylogenetic tree of the CMGC group, focused on branches with strong bootstrap support between *Plasmodium* and human sequences**.** A small number of *Plasmodium* kinases within the CMGC group exhibited homology to human kinases. These kinases were *Plasmodium* kinases PfCLK3, PfSRPK1, PfMAPK1, PfCKα and PfGSK3 (denoted with a red circle). The associated strong bootstrap support (> 80) has been coloured blue, along with the branches to the human homologs. Bootstrap values are listed on the associated branches (when > 30), *P. falciparum* kinases are highlighted in blue, *P. vivax* kinases are highlighted in red

Each ePK group (including FIKK and orphan kinases) was assessed to determine if any particularly strong bootstrap support for homology between the *Plasmodium* and human protein kinases was observed (Supplementary data 2). 37 *P. falciparum* kinases, including the 21 FIKKs, do not have any bootstrap support to any human sequence (primarily the FIKKs). and a further 46 display bootstrap support less than 50 to any human homolog. These kinases with minimal similarity to human ePKs represent attractive targets for selective intervention. Surprisingly (in view of the divergent evolutionary paths of Alveolates and Metazoans), a number of noteworthy homologies were identified across most groups. The *Plasmodium* kinases which exhibited bootstrap support to human kinase/s greater than 50 are listed in Table 2, along with their human homolog(s). Table 2 notes that of the 16 *Plasmodium* kinases with bootstrap support above 50 to a human homolog(s), 10 of these belong to the CMGC group. Further, all *Plasmodium* kinases with bootstrap support values over 75, to a human homolog, belong to the CMGC group. Most notable are the *Plasmodium* kinase, cyclin-dependent-like kinase 3 (CLK3), serine/arginine protein kinase 1 (SRPK1), Casein kinase 2 alpha subunit (CK2a), Mitogen activated protein kinase 1 (MAPK1) and Glycogen synthase kinase 3 (GSK3) (Fig. 4; a tree with the entire CMGC family, along with the CMGC kinases of *P. vivax*, is available in Supplementary Fig. 2).

**Table 2:** List of *Plasmodium* kinases with bootstrap support greater than 50 to a human homolog

*P. falciparum* ID	*P. vivax* ID	Name	Kinase group	closest human homolog/s	bootstrap support
PF3D7_1114700	PVP01_0915400	CLK3	CMGC	PRPF4B	100
PF3D7_0302100	PVP01_0302100	SRPK1	CMGC	SRPK1, SRPK2, SRPK3	99
PF3D7_1108400	PVP01_0909200	CK2a	CMGC	CSNK2A1, CSNK2A2, CSNK2A3	99
PF3D7_1431500	PVP01_1431500	MAPK1	CMGC	MAPK15	97
PF3D7_0312400	PVP01_0827800	GSK3	CMGC	GSK3A, GSK3B	87
PF3D7_1356900	PVP01_1115000	PK5	CMGC	CDK5, CDK14, CDK15, CDK16, CDK17, CDK18	84
PF3D7_0708300	PVP01_0108600	BUD32	Orphan	TP53RK	68
PF3D7_1121300	PVP01_0921900	TKL2	TKL	IRAK1, IRAK2, IRAK3, IRAK4	68
PF3D7_1228300	PVP01_1446500	NEK1	NEK	NEK2	67
PF3D7_1445400	PVP01_1261600	CLK1	CMGC	CLK1, CLK2, CLK3, CLK4	61
PF3D7_0415300	PVP01_0521800	CRK3	CMGC	CDK9, CDK12, CDK13	60
PF3D7_0821100	PVP01_0513200	PK1	CMGC	GSK3A, GSK3B	57
PF3D7_1316000	PVP01_1416900	-	CMGC	GSK3A, GSK3B	57
PF3D7_1246900	PVP01_1464000	PKB	AGC	SGK1, SGK2, SGK3, AKT1, AKT2, AKT3, PRKCI, PRKCZ, PRKCH, PRKCE, PRKCB, PRKCG, PRKCA, PRKCQ, PRKCD, PKN1, PKN2, PKN3	56
PF3D7_1136500	PVP01_0937400	CK1	CK1	CSNK1A1, CSNK1A1L	55
PF3D7_1436600	PVP01_1312900	PKG	AGC	PRKG1, PRKG2	53

**CLK3** (PF3D7_1114700), belongs to the CLK family of protein kinases, which in mammalian cells, facilitate phosphorylation of splicing factors [45]. In *Plasmodium*, PfCLK3 is essential during asexual blood stage development [46]. PfCLK3 has previously been assigned to the PRP4 subfamily of dual-specificity tyrosine-regulated kinases (DYRK) [38]. Our phylogenetic analysis confirms this finding, and further reports the striking similarity between PfCLK3 and *Hs*PRPF4B (bootstrap support = 100). **SRPK1** (PF3D7_0302100), was initially considered to belong to the CLK family, however, it was reclassified as a SRPK following functional analysis [47]. SRPKs are closely related to the CLKs, and in mammalian cells, have a number of complex functions including mRNA processing and nuclear import (reviewed in [48]). In *Plasmodium*, PfSRPK1 (previously known as PfCLK4) is essential during asexual blood stage development [46]. Here we can confirm the homology of *Pf*SRPK1 as the kinase clusters closely with the human SRPK1-3 (Bootstrap support = 99). Interestingly, PfSRPK2 branches away before the SRPK and CLK division in the tree and is likely to have evolved from the precursor gene that gave rise to SRPKs and CLKs in the Opisthokont lineage. **CK2α** (PF3D7_1108400), and **GSK3** (PF3D7_0312400) are present in all examined apicomplexan species. In *Plasmodium*, PfCK2α and PfGSK3 are essential for blood stage development [46]. PfCK2α is homologous to human CSNK2A1-3 (Bootstrap support = 99) and PfGSK3 shows homology to human GSK3α/β. Interestingly, *Plasmodium* PK6, PK1, PF3D7_1316000 and GSK3 cluster in the same branch as Human GSKα/β, which could suggest these three kinases derive from a common gene.

The Mitogen activation protein kinase family forms two relatively tight clusters of protein kinases within the CMGC group (Fig. 2). MAPKs typically function as part of a three-tiered MAPK cascade, where a MAP3K phosphorylates a MAP2K which in turn phosphorylates a MAPK. **PfMAPK1** (**Pfmap-1**, PF3D7_1431500) clusters closely with human MAPK15 (ERK7, bootstrap support = 97), forming a clade that branches away from the majority of the MAPKs early in the tree (Figs. 2 and 4). In humans, MAPK15 (ERK7) is an atypical MAPK that is activated by auto-phosphorylation rather than in the context of a classical 3-tier pathway [49]. In *P. falciparum* MAPK1 has been shown to be dispensable during erythrocytic development and for sporogony in the mosquito [46, 50]. Curiously however, the other MAPK encoded by *P. falciparum*, **MAPK2** (**pfmap-2**, PF3D7_1113900) has been demonstrated to be elevated in MAPK1 knockouts, suggesting the parasite is able to adaptively compensate for reduced MAPK1 levels [50]. PfMAPK2 clusters within one of the primary branches of the human MAPK family (MAPK1/3/4/6/7 and NLK) (bootstrap support = 86). Further, within this family PfMAPK2 clusters closest to human MAPK1/3/4 and 6 (bootstrap support = 35, Fig. 4). Despite a clear homolog to the above-mentioned family of MAPKs, *Plasmodium* does not possess a MAP2K orthologue to phosphorylate and activate PfMAPK2. In fact, both *P. falciparum* and *P. vivax* only encodes a single member of the STE group (containing the MAP2Ks), which does not cluster closely with any specific kinase (Supplementary Fig. 3). Whether these enzymes function in pathways that implicate the MAPKs remains to be determined.

### Comparison of the *P. falciparum* and *P. vivax kinome*

**Fig. 5:**
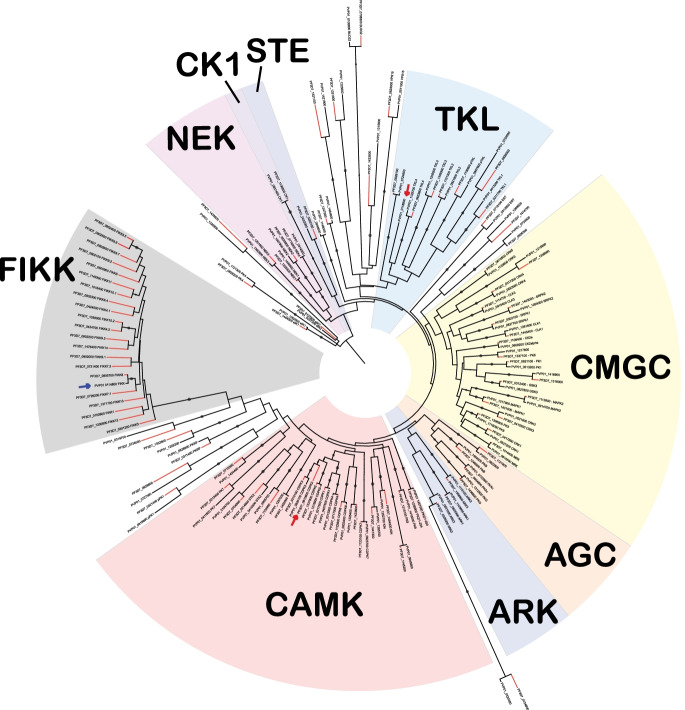
Comparative Phylogenetic tree of *Plasmodium vivax* and *Plasmodium falciparum*. Phylogenetic tree indicating the kinases shared and unique to each of the two species, *P. falciparum* (red branches), *P. vivax* (black branches). Red arrow indicates the kinases PVP01_0118800 and PF3D7_0610600 (CDPK2) which do not have an equivalent kinase in the other species. Blue arrow indicates PVP01_0114800, the only member of the FIKK family that *P. vivax* encodes. See Fig. 1 legend for details regarding the assembly and construction of the phylogenetic tree. Typical protein kinase families were annotated using Adobe illustrator along with the Aurora kinase family (ARK) and the Apicomplexan-specific kinase family FIKK. Orphan, or ‘other’ kinases are largely unassigned to families (white background). Bootstraps values above 50 are represented as circles on the associated branches, larger circles indicate higher bootstrap values, note these values relate to the full tree illustrated in Fig. 2

To directly compare the kinomes of *P. falciparum* and *P. vivax*, while preserving the phylogenetic tree structure, all *H. sapiens* branches were removed from the tree (Fig. 5). As alluded to above, the kinomes of *P. vivax* and *P. falciparum*, despite their evolutionary distance, are very similar, with almost all kinases having a clear orthologue in the other species. There are only three distinct cases where no orthologue was observed (red arrows in Fig. 5): first, as previously reported [11], there is only one FIKK encoded by the *P. vivax* genome, versus a paralogous group of 21 sequences in *P. falciparum.* Second, PVP01_0118800, which belongs to the TKL family, does not have an orthologue in *P. falciparum*. Third, PfCDPK2, from the CAMK family does not have an orthologue in *P. vivax*.

**Fig. 6:**
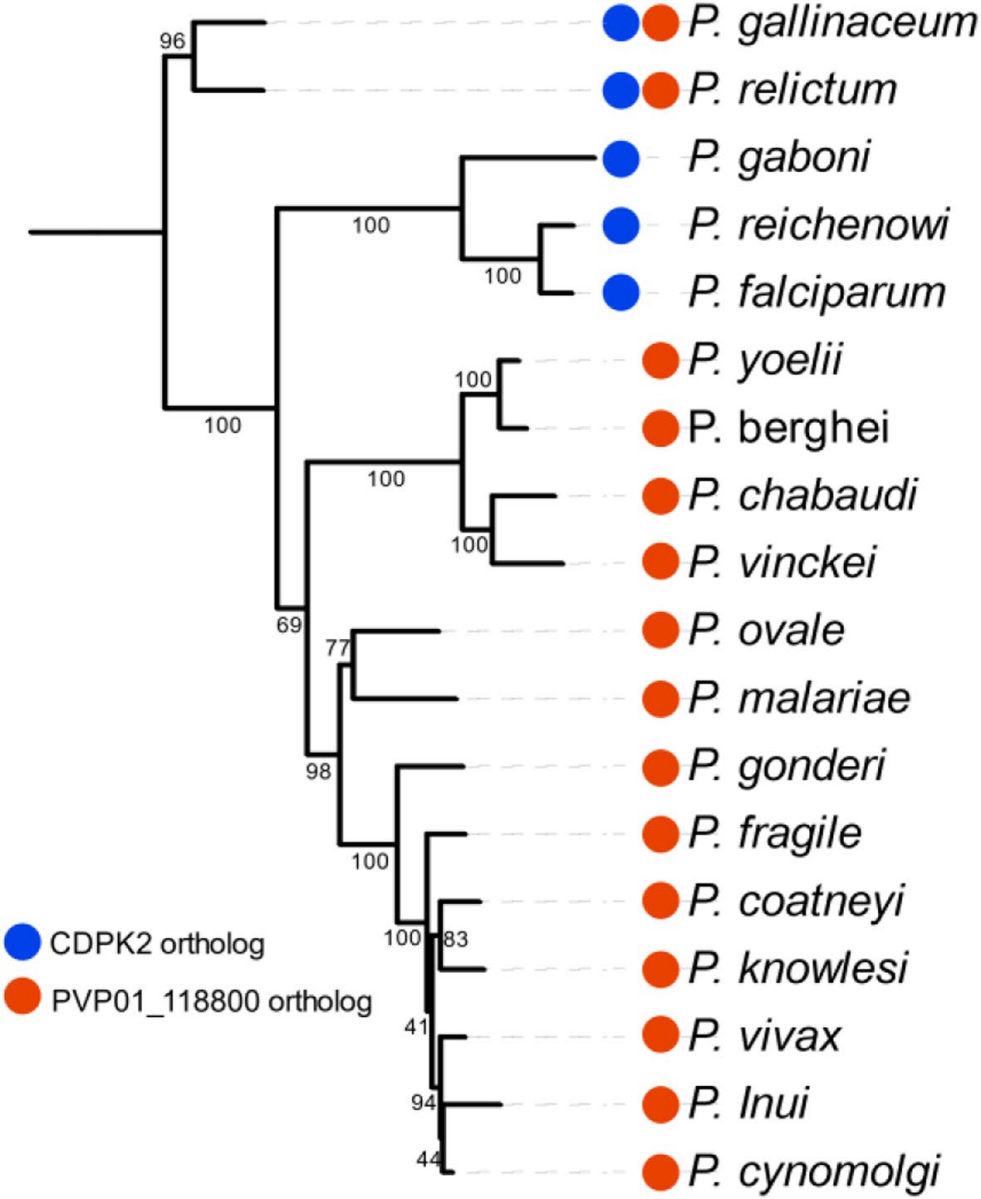
Phylogenetic tree of *Plasmodium spp*. illustrating CDPK2 and PVP01_118800 orthologs**.** Tree assembled using the mitochondrial genomes of each species (see methods for details). A gene tree was inferred through the RAxML analysis using the ‘best tree’ and rendered with the tree of life webserver (iTOL) [44]. CDPK2 and PVP01_118800 orthologs were identified through blast searches using the kinase domains. *Plasmodium* species which have orthologs to CDPK2 are indicated by the blue circle, while species with an ortholog of PVP01_118800 are indicated by the red circle. Bootstrap support for the gene tree is indicated on each of the branches, only values above 40 are displayed

To understand which clades within the *Plasmodium* genus possess an orthologue of CDPK2, we aligned the kinase domains of all known CDPKs encoded by six distinct species; *P. falciparum*, *P. vivax*, *Plasmodium knowlesi*, *Plasmodium berghei*, *Plasmodium gaboni* and *Plasmodium gallinaceum* (see "Methods" section). These sequences were assessed using RAxML [41, 42] and a gene tree inferred from the results [44]. From the gene tree it is clear that CDPK2 is significantly different from its next closest homologue in the *Plasmodium* genus (CDPK3) (Supplementary Fig. 10). To more extensively assess which species in the genus *Plasmodium* encoded an orthologue of CDPK2 we completed a BLASTP search using the kinase domain of PfCDPK2. We compared the species that contained an orthologue of CDPK2 to the mitochondrial genome phylogeny for the *Plasmodium spp.* on PlasmoDB [17] (see "Methods" for details). This identified that the bird-infecting *Plasmodium gallinaceum* and *Plasmodium relictum*, as well as species in the *Laverania* lineage (*Plasmodium gaboni*, *Plasmodium rechenowi* and *P. falciparum)* all contain an orthologue of CDPK2, while species from the murine parasite clades and the other (non-Laveranian) primate-infecting parasites lineages do not **(**Fig. 6**)**. This is consistent with a whole-genome-based phylogeny suggesting that the Laverania have been founded by a single *Plasmodium* species switching from birds to African great apes (or vice versa, see below), and suggest that CDPK2 has been lost in all other *Plasmodium* clades, or gained after the split between the clades [51].

### Regulatory subunits of kinases

Protein kinase regulatory subunits do not themselves have protein kinase activity, but are essential in the regulation of a select few protein kinases. Casein kinase 2 (CK2), which belongs to the CMGC group, forms a homo- or hetero-tetramer structure comprised of two regulatory subunits and two catalytic subunits [52]. *P. falciparum* encodes a single CK2 catalytic subunit (PF3D7_1108400) and two different regulatory subunits (PF3D7_1103700 and PF3D7_1342400) [34]. BLASTP searches confirmed that *P. vivax* encodes orthologs to each of these subunits (CK2 catalytic subunit: PVP01_0909200 and regulatory subunits: PVP01_0904500, PVP01_1212400) and no other. Protein Kinase A (PKA) belongs to the AGC ePK group, and, similar to CK2, the human holoenzyme is structured as a tetrameter of two catalytic subunits and two regulatory subunits; cAMP binding to the regulatory subunits results in the release of the active catalytic subunits [53]. *P. falciparum* has previously been reported to encode a single PKA regulatory (PF3D7_1223100) and a single catalytic subunit (PF3D7_0934800) [54], and the same is true for *P. vivax* (regulatory subunit: PVP01_0733500; catalytic subunit: PVP01_0733500). Lastly, cyclins are a diverse family of proteins that contain a conserved 5-helix bundled region known as the cyclin box, which enables binding to cyclin-dependent kinases (CDKs), stimulating their activity and hence playing a major role in cell cycle control [55]. *P. falciparum* encodes 3 readily identifiable cyclins, CYC1 (PF3D7_1463700), CYC3 (PF3D7_0518400) and CYC4 (PF3D7_1304700) as well as CYC2 (PF3D7_1227500), which appears to be more distantly related [56, 57]. We completed an HMM search using both the PFAM IDs PF086134 (Cyclin) and PF00134 (Cyclin_N) profiles, which revealed the presence of a fifth, previously unreported, putative cyclin in *P. falciparum* (PF3D7_0605500). Additionally, the same search identified four possible cyclins in *P. vivax*, (PVP01_1015500, PVP01_1243100, PVP01_1143400 and PVP01_1405600). Phylogenetic analysis of the cyclin box (a highly conserved sequence among cyclins) showed that PVP01_1243100, PVP01_1015500 and PVP01_1405600 are orthologous to PfCYC1, PfCYC3 and PfCYC4 respectively. Lastly, PVP01_1143400 and PF3D7_0605500, had not been identified previously and appear to be distantly related to Human Cyclin A (Supplementary Fig. 11). Further refined phylogenetic analysis and functional validation of these putative cyclins are warranted.

## Concluding remarks

In this study we generated a complete human kinome comparison to *P. falciparum* and *P. vivax*, enabling the first comprehensive assessment of homologues protein kinases between an Apicomplexan parasite and its primary host. The striking kinome conservation observed across the evolutionarily distance species of *P. vivax* to *P. falciparum* together with previous studies of *P. berghei* [58] and earlier kinome assemblies of *P. falciparum* confirm that there is clear pressure for *Plasmodium spp.* to maintain the overwhelming majority of its kinome [11, 38]. Though there are examples of clade-specific gene loss, such as CDPK2 and PVP01_118800 reported here, the vast majority of kinases remain highly conserved. In *P. falciparum*, *Pf*CDPK2 is critical for male gametogenesis [59]; therefore, it is likely that this function is fulfilled by another CDPK in the species where it is absent. The homology observed between CDPK2 and CDPK3 suggest that CDPK3 may fulfil this function, although this remains to be demonstrated (Supplementary Fig. 10). PVP01_118800 belongs to the TKL group and our kinome comparison indicates that it is most closely related to TKL4 of both *P. falciparum* and *P. vivax*, with a strong bootstrap support of 67 **(**Figs. 2 and 5**)**. A BLASTP search using PVP01_118800’s kinase domain as a query, indicated that an orthologue exists in all *Plasmodium* species with annotated genomes, except for species within the *Laverania* clade; this supports the hypothesis that the *Laverania* lineage results from a transfer from the bird-infecting parasites to the great apes, rather than the reverse [51], and that PVP01_118800 homologs were lost after passage of the *Laverania* founding species from birds to great apes **(**Fig. 6**)**.

## Methods

### PIKK and cyclin phylogeny

The *Plasmodium falciparum* and *vivax*, PIKK and cyclin family of proteins were collected from PlasmoDB v50b [17] using the Pfam IDs of PF00134 and PF08613 (Cyclins) and PF00454 (PIKKs) *P. falciparum* reference genome version GCA_000002765.3 [60, 61], *P. vivax* reference genome version GCA_900093555.2 [62]. To determine the validity of their assignment to the PIKK or Cyclin families, we created a HMM profile of the Cyclin box and atypical kinase domain (PIKKs) using a seed of the respective Pfam IDs available at pfam.xfam.org/ [63], as input into HMMER [22]. Following HMM profile identification the conserved sequences of the *Plasmodium* Cyclin box and atypical kinase domain of the PIKKs were aligned using ClustalOmega [23] and manually corrected in Jalview [24] before RAxML phylogenetic inference [41, 42]. RAxML settings: maximum-likelihood rapid bootstrap with the PROTgamma substitution model LG4M, and AutoMRE. The gene tree was rendered using the webserver iTOL [44].

### Plasmodium protein kinase sequences

The *P. falciparum* sequences were collected from an earlier alignment [8], and *P. vivax* sequences were obtained by searching PlasmoDB v50b [17], *P. falciparum* reference genome version GCA_000002765.3 [62] (PMID: 31080894), *P. vivax* reference genome version GCA_900093555.2 [60, 61]. The predicted *P. vivax* proteome in PlasmoDB v50b was searched using the term “kinase”. The resultant list was further refined to only include, (i) sequences containing a Pfam ID of PF00069 or PF07714 (Protein kinase domain and protein tyrosine kinase, respectively) and (ii) sequences which contained the phrase “protein kinase” in the sequences annotated ‘Product description’ data.

### NEK HMM profile creation

Kinomer [25], the HMM profile databased used in this study did not include a profile for NEK family. The human protein kinase MSA which was the backbone of this study designated protein kinases into the NEK family. To be consistent with this and to enable comparison between species, we developed a NEK profile using HMMER’s hmmbuild function (HMMER 2.3.2) [22], amending the Kinomer HMM profile with our NEK profile. Our NEK profile was comprised of 21 unique NEK sequences from 11 different organisms, which spanned the primary branches within the NEK family [29] (sequences available in Supplementary Data 3).

### Multiple sequence alignment

The MSA developed for the human kinome contained 17 highly conserved blocks/segments interspersed with unaligned blocks of amino acids of variable length. The 17 aligned segments were defined using the structure of Human Aurora kinase A [16] and are similar to the regions initially defined by the original alignment published in 1988 by Hanks et al. [64]. These regions are named as follows; B1N, B1C, B2, B3, HC, B4, B5, HD, HE, CL, ALN, ALC, HF, FL, HG, HH and HI (see [16] for more details). Each of the *Plasmodium* kinase domains were aligned according to these 17 segments using the human MSA as a reference. For each *Plasmodium* kinase, the HMM profile was used to determine the closest related human kinases. For *Plasmodium* kinase domains designated as orphans, the nearest homologue was identified using PSI-BLAST and used to guide the alignment, leading to the definition of a 230- column amino acid sequence (one amino acid per column). The complete MSA contained a total of 3183 columns due to a number of large extensions in some *Plasmodium* kinases, notably SRPK1, PK1 and EST. The complete MSA of the *P. falciparum* and *P. vivax* kinases domains is available as Supplementary data 4.

### Phylogenetic relationship determination

The phylogeny relationships were determined in the RAxML GUI 2.0 [41, 42], using the 17 conserved sections of the MSA (230 columns) of the kinase domains of *H. sapiens*, *P. falciparum* and *P. vivax* as defined by [16]. The following parameters were used in RAxML: maximum-likelihood rapid bootstrap with the PROTgamma substitution model LG4M, and AutoMRE. A total of 200 bootstrapping runs was performed to meet the AutoMRE requirement. The gene tree was rendered using the webserver iTOL [44] before further annotations of the kinase families in Adobe illustrator. The definition of the borders for each family was guided by the HMM profiles, the tree structure and the defined family assignment reported for each of the human kinases [16].

### Plasmodium mitochondrial genome phylogeny

The mitochondrial genome of *P. gallinaceum*, *P. relictum*, *P. gaboni*, *P. rechenowi*, *P. falciparum*, *P.yoelii*, *P.berghei*, *P. chabaudi*, *P. vinckei*, *P. ovale*, *P. malariae*, *P. gonderi*, *P.fragile*, *P. coatneyi*, *P. knowlesi*, *P. vivax*, *P. Inui* and *P. cynomolgi* were obtained from GenBank [65] and aligned using ClustalOmega [23], and imported into Jalview [24] and manually corrected. The MSA was imported into RAxML Gui 2.0 [42], and the following parameters input: maximum-likelihood rapid bootstrap with the gamma substitution model GTR with proportion of invariant sites through ML estimate (+ I), with AutoMRE. A gene tree was inferred through the RAxML analysis using the ‘best tree’ and rendered with the interactive tree of life webserver (iTOL) [44].

## Supplementary Information

**Additional file 1:** **Supplementary data 1. **HMM of the protein kinase family/group assignment for each of the typical protein kinases investigated here. The protein kinase family/group HMM profiles were acquired from the Kinomer database [29], and the NEK HMM profile was generated in this study (See Supplementary data 1).

**Additional file 2: Supplementary data 2.** Full table of protein kinase homology comparison between the *Plasmodium* species of this study and *Homo sapiens*, using the RAxML bootstrapping support values. 

**Additional file 3:** **Supplementary data 3. **Alignment of the protein kinase domains of diverse subset of NEKs across 11 different species. This alignment was used to generate a HMM profile for this family used in this study.

**Additional file 4: Supplementary data 4.** Complete Multiple sequence alignment for the typical protein kinases of Homo sapiens, *Plasmodium falciparum* and *Plasmodium vivax*. Each sequence is aligned into the 17 highly conserved regions of the protein kinase domain. The sequences of the 17 aligned blocks are capitalised, with the extension regions between these conserved blocks in lowercase and left-justified.

**Additional file 5: Supplementary Figure 1.** Phylogenetic tree comparing the members of the PI3/PI4 kinases in *Plasmodium falciparum* (highlighted blue) and *Plasmodium vivax* (highlighted red) to a seed of sequences representative of the family (Pfam ID PF00454) available at pfam.xfam.org/ [57]. Bootstrap support greater than 50 are indicated on the respective branches. RAxML settings: maximum-likelihood rapid bootstrap with the PROTgamma substitution model LG4M, and AutoMRE. The gene tree was rendered using the webserver iTOL [53].

**Additional file 6:Supplementary Figures 2 - 8.** The kinase families/groups identified in Figure 2 are depicted in the form of phylogenetic trees: CMGC (Sup Fig 2), STE (Sup Fig 3), TKL (Sup Fig 4), CK1 and NEK (Sup Fig 5), CAMK (Sup Fig 6), ARK (Sup Fig 7), AGC (Sup Fig 8). *Plasmodium falciparum* kinases (highlighted blue) and *Plasmodium vivax* kinases (highlighted red). Bootstrap support greater than 50 are indicated on the respective branches.

**Additional file 7:** **Supplementary Figure 9.** Sequence logos for the 17 highly conserved regions of the protein kinase domain for *Homo sapiens* and *Plasmodium*(*P. falciparum,P. vivax*).

**Additional file 8:** **Supplementary Figure 10.** Phylogenetic tree comparing the members of the CDPK family in *Plasmodium falciparum,Plasmodium vivax, Plasmodoum knowlesi, Plasmodium berghei, Plasmodium gallinaceum and Plasmodium gaboni*. Bootstrap support greater than 50 are indicated on the respective branches (as circles). RAxML settings: maximum-likelihood rapid bootstrap with the PROTgamma substitution model LG4M, and AutoMRE. The gene tree was rendered using the webserver iTOL [53].

**Additional file 9:** **Supplementary Figure 11.** Phylogenetic tree comparing the members of the Cyclin family in *Plasmodium falciparum *(highlighted blue) and *Plasmodium vivax* (highlighted red) to a seed of sequences representative of the family (Pfam IDs PF00134 and PF08613) available at pfam.xfam.org/ [57]. Bootstrap support greater than 50 are indicated on the respective branches. RAxML settings: maximum-likelihood rapid bootstrap with the PROTgamma substitution model LG4M, and AutoMRE. The gene tree was rendered using the webserver iTOL [53].

## Data Availability

All data are available in the Supplementary datasets provided.

## Abbreviations

ePK: Eukaryotic protein kinase
aPK: Atypical protein kinase
CAMK: Calcium/Calmodulin regulated kinases
CMGC: Comprises the CDK, MAPK, GSK, and CLK families
CK1: Casein Kinase I
NEK: NimA related kinases
AGC: Comprises the protein kinase A/G/C families
RGC: Receptor Guanylate Cyclases
TKL: Tyrosine Kinase Like
TK: Tyrosine Kinase
ARK: Aurora Kinases
OPK: Orphan Protein Kinase
MSA: Multiple Sequence Alignment
CL: Catalytic Loop
ALN: Activation Loop N-terminal
ALC: Activation Loop C-terminal
