# Supplementary figures and images for: Comparative analysis of the kinomes of Plasmodium falciparum, Plasmodium vivax and their host Homo sapiens
Source: BMC Genomics. 2022 Mar 26;23:237. doi: 10.1186/s12864-022-08457-0 (PMC8960227; doi:10.1186/s12864-022-08457-0)

# PI3/PI4 Kinase

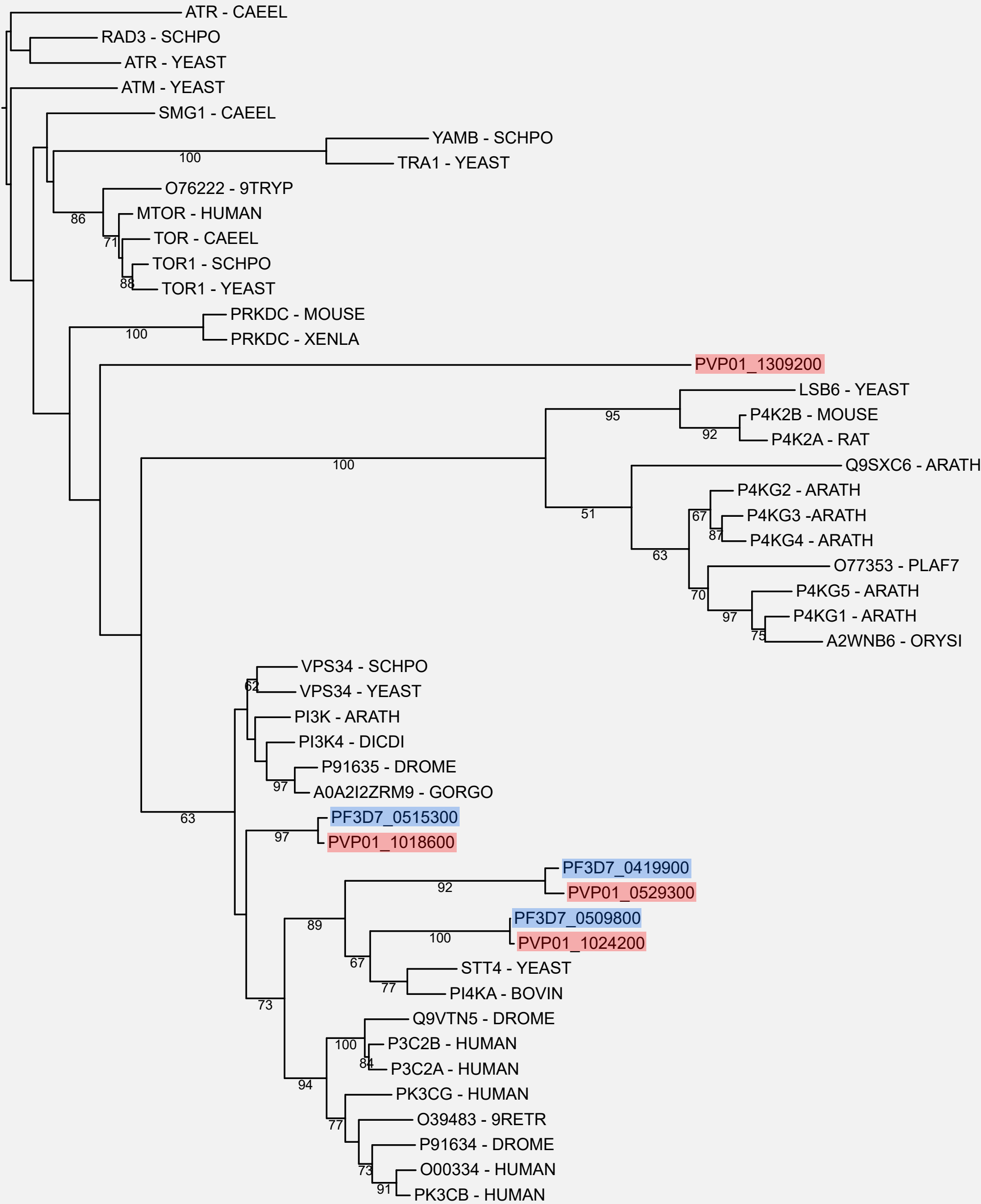

Supplement: Supplementary file 5 — Additional file 5: Supplementary Figure 1. Phylogenetic tree comparing the members of the PI3/PI4 kinases in Plasmodium falciparum (highlighted blue) and Plasmodium vivax (highlighted red) to a seed of sequences representative of the family (Pfam ID PF00454) available at pfam.xfam.org/ [57]. Bootstrap support greater than 50 are indicated on the respective branches. RAxML settings: maximum-likelihood rapid bootstrap with the PROTgamma substitution model LG4M, and AutoMRE. The gene tree was rendered using the webserver iTOL [53]. [file 12864_2022_8457_MOESM5_ESM.pdf]

# Cyclins

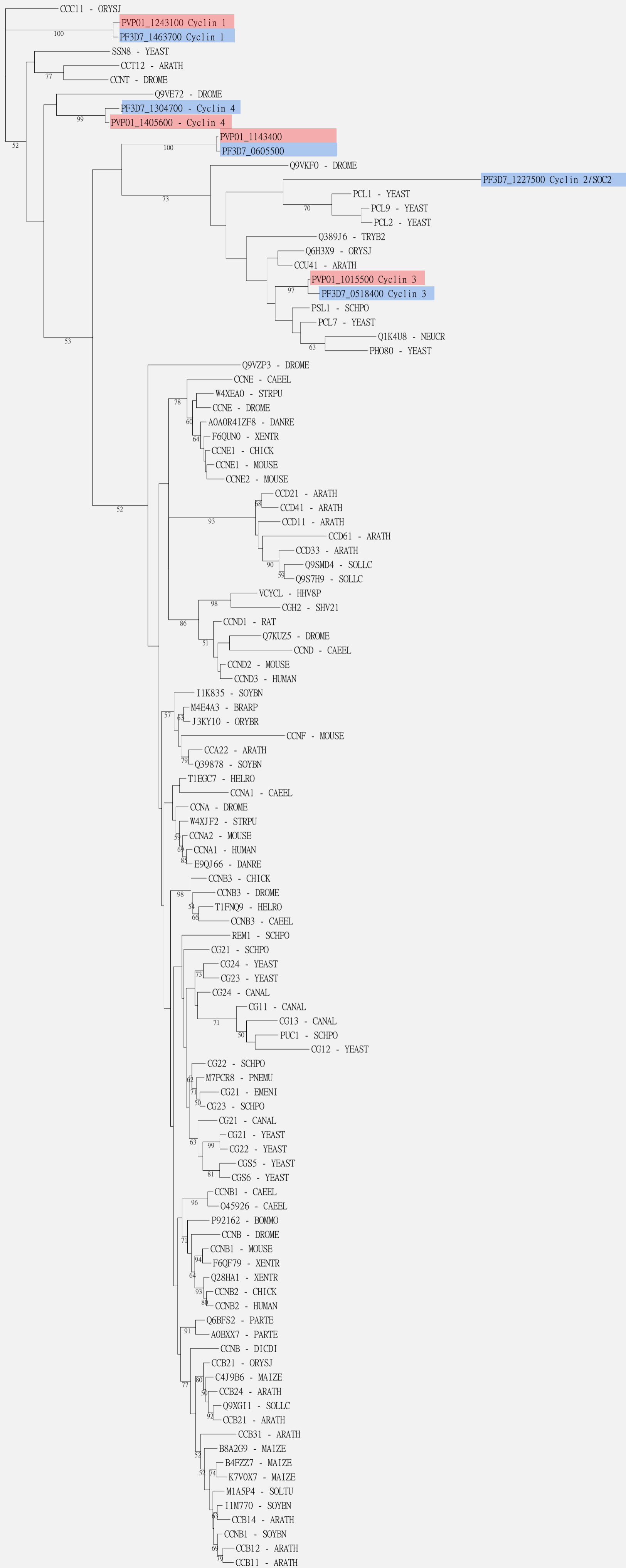

Supplement: Supplementary file 7 — Additional file 7: Supplementary Figure 9. Sequence logos for the 17 highly conserved regions of the protein kinase domain for Homo sapiens and Plasmodium(P. falciparum,P. vivax). [file 12864_2022_8457_MOESM7_ESM.pdf]

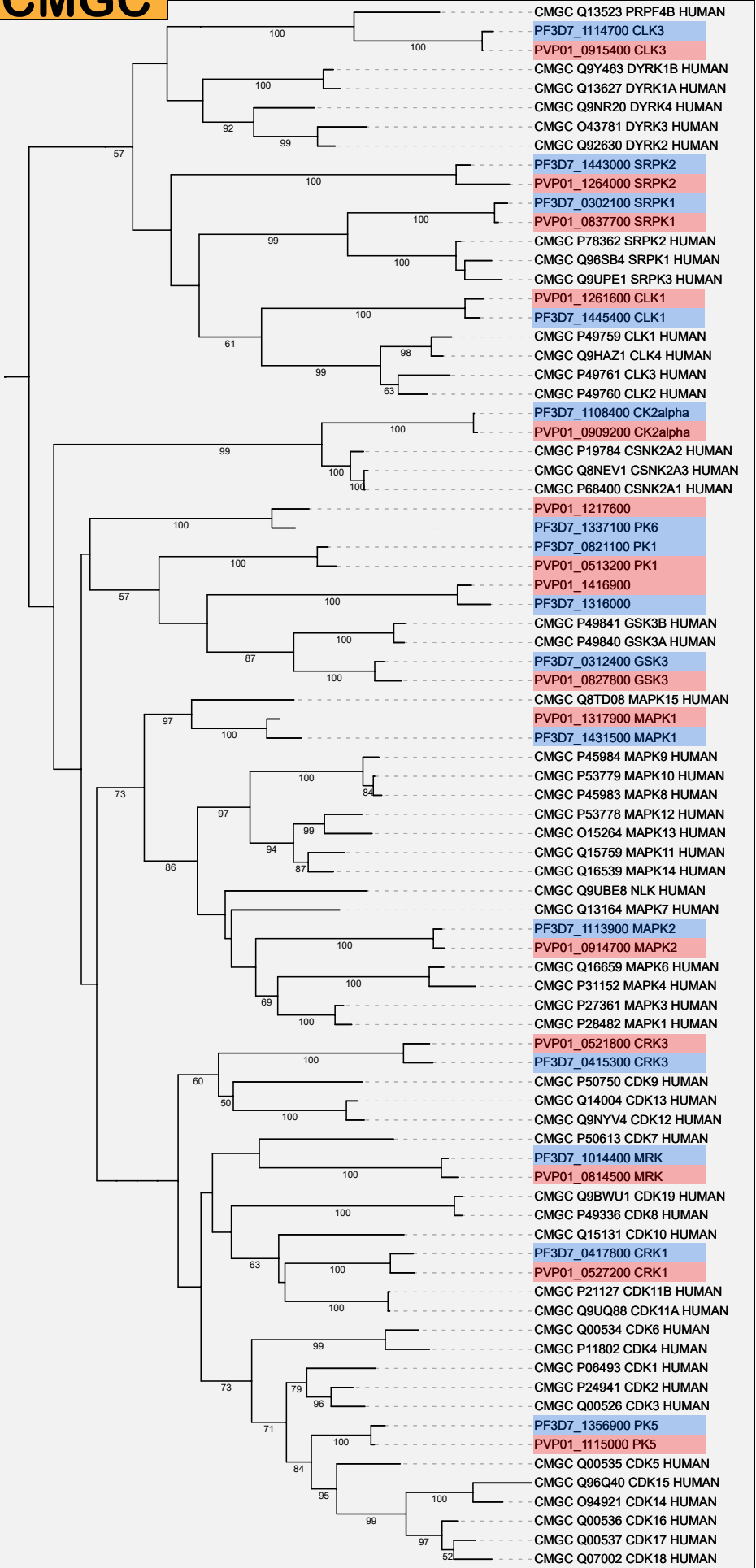

Supplement: Supplementary file 8 — Additional file 8: Supplementary Figure 10. Phylogenetic tree comparing the members of the CDPK family in Plasmodium falciparum,Plasmodium vivax, Plasmodoum knowlesi, Plasmodium berghei, Plasmodium gallinaceum and Plasmodium gaboni. Bootstrap support greater than 50 are indicated on the respective branches (as circles). RAxML settings: maximum-likelihood rapid bootstrap with the PROTgamma substitution model LG4M, and AutoMRE. The gene tree was rendered using the webserver iTOL [53]. [file 12864_2022_8457_MOESM8_ESM.pdf]

# STE

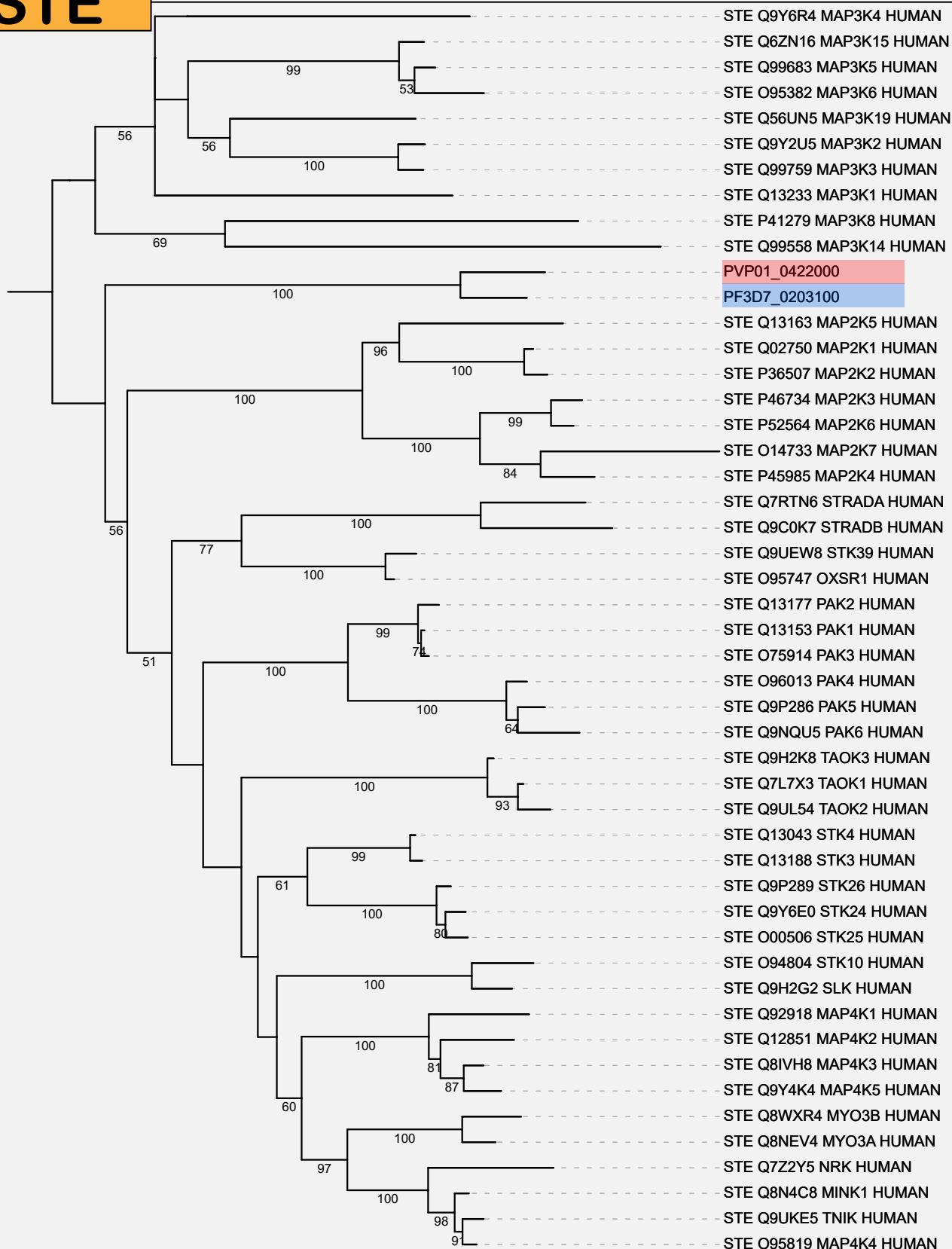

Supplement: Supplementary file 9 — Additional file 9: Supplementary Figure 11. Phylogenetic tree comparing the members of the Cyclin family in Plasmodium falciparum (highlighted blue) and Plasmodium vivax (highlighted red) to a seed of sequences representative of the family (Pfam IDs PF00134 and PF08613) available at pfam.xfam.org/ [57]. Bootstrap support greater than 50 are indicated on the respective branches. RAxML settings: maximum-likelihood rapid bootstrap with the PROTgamma substitution model LG4M, and AutoMRE. The gene tree was rendered using the webserver iTOL [53]. [file 12864_2022_8457_MOESM9_ESM.pdf]
