# Supplementary material for: Comparative analysis of the kinomes of Plasmodium falciparum, Plasmodium vivax and their host Homo sapiens
Source: BMC Genomics. 2022 Mar 26;23:237. doi: 10.1186/s12864-022-08457-0 (PMC8960227; doi:10.1186/s12864-022-08457-0)
Supplement: Supplementary file 6 — Additional file 6:Supplementary Figures 2 - 8. The kinase families/groups identified in Figure 2 are depicted in the form of phylogenetic trees: CMGC (Sup Fig 2), STE (Sup Fig 3), TKL (Sup Fig 4), CK1 and NEK (Sup Fig 5), CAMK (Sup Fig 6), ARK (Sup Fig 7), AGC (Sup Fig 8). Plasmodium falciparum kinases (highlighted blue) and Plasmodium vivax kinases (highlighted red). Bootstrap support greater than 50 are indicated on the respective branches. [file 12864_2022_8457_MOESM6_ESM.pdf]

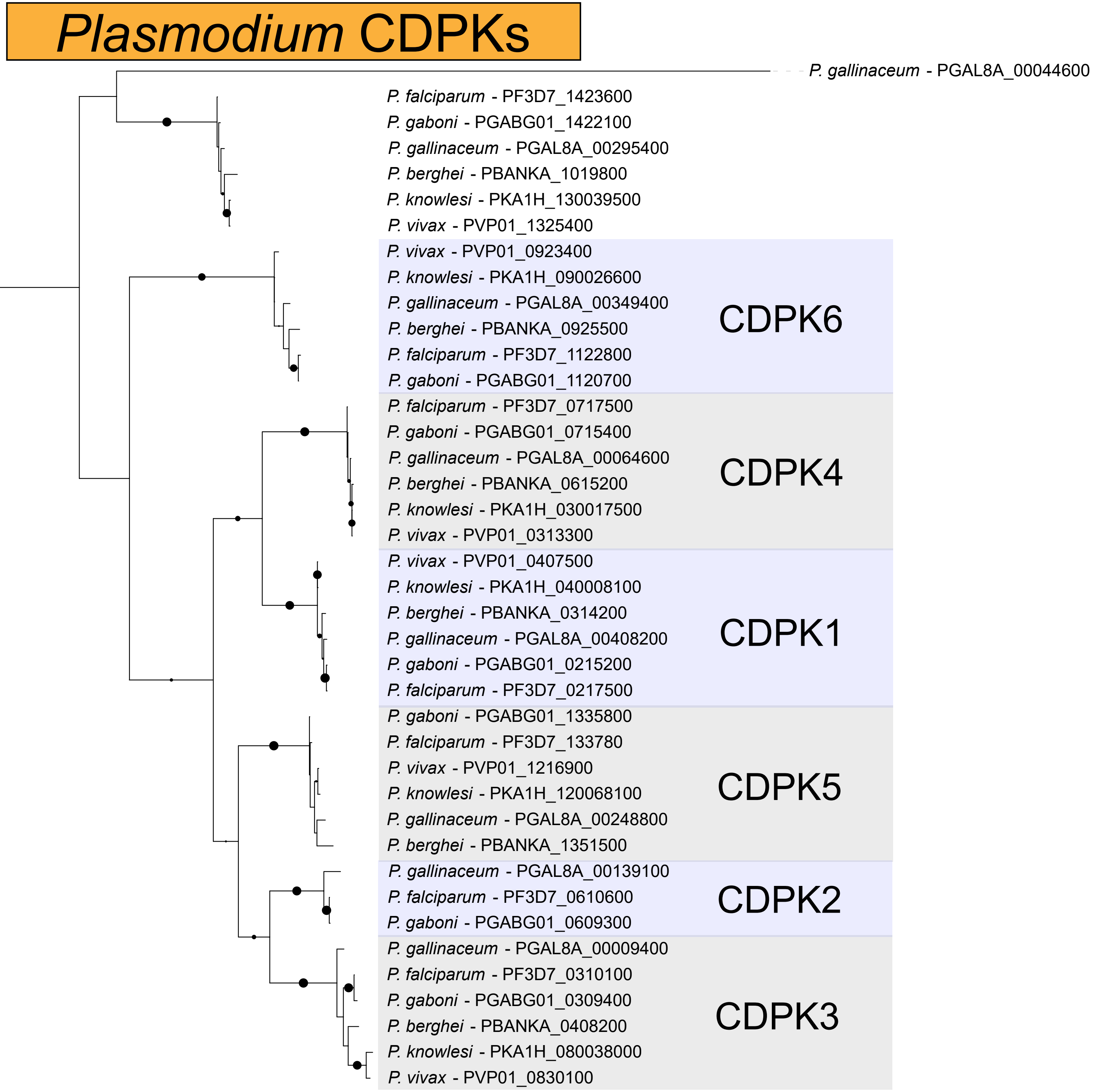

Plasmodium CDPKs
P. gallinaceum - PGAL8A_00044600
P. falciparum - PF3D7_1423600
P. gaboni - PGABG01_1422100
P. gallinaceum - PGAL8A_00295400
P. berghei - PBANKA_1019800
P. knowlesi - PKA1H_130039500
P. vivax - PVP01_1325400
P. vivax - PVP01_0923400
P. knowlesi - PKA1H_090026600
P. gallinaceum - PGAL8A_00349400
P. berghei - PBANKA_0925500
P. falciparum - PF3D7_1122800
P. gaboni - PGABG01_1120700
CDPK6
P. falciparum - PF3D7_0717500
P. gaboni - PGABG01_0715400
P. gallinaceum - PGAL8A_00064600
P. berghei - PBANKA_0615200
P. knowlesi - PKA1H_030017500
P. vivax - PVP01_0313300
CDPK4
P. vivax - PVP01_0407500
P. knowlesi - PKA1H_040008100
P. berghei - PBANKA_0314200
P. gallinaceum - PGAL8A_00408200
P. gaboni - PGABG01_0215200
P. falciparum - PF3D7_0217500
CDPK1
P. gaboni - PGABG01_1335800
P. falciparum - PF3D7_133780
P. vivax - PVP01_1216900
P. knowlesi - PKA1H_120068100
P. gallinaceum - PGAL8A_00248800
P. berghei - PBANKA_1351500
CDPK5
P. gallinaceum - PGAL8A_00139100
P. falciparum - PF3D7_0610600
P. gaboni - PGABG01_0609300
CDPK2
P. gallinaceum - PGAL8A_00009400
P. falciparum - PF3D7_0310100
P. gaboni - PGABG01_0309400
P. berghei - PBANKA_0408200
P. knowlesi - PKA1H_080038000
P. vivax - PVP01_0830100
CDPK3
